# Correction: Aggregating multiple test results to improve medical decision-making

**DOI:** 10.1371/journal.pcbi.1013347

**Published:** 2025-08-04

**Authors:** Lucas Böttcher, Maria R. D’Orsogna, Tom Chou

There is an error in reference 75. The correct reference is: Böttcher L, Klingebiel R. Organizational selection of innovation. Organization Science. 2025;36,387–410.
