# Supplementary material for: Aggregating multiple test results to improve medical decision-making
Source: PLoS Comput Biol. 2025 Jan 7;21(1):e1012749. doi: 10.1371/journal.pcbi.1012749 (PMC11741652; doi:10.1371/journal.pcbi.1012749)
Supplement: Attachment — Submitted filename: PLOS_COMP_BIO_PCOMPBIOL-D-24-00885.pdf [file pcbi.1012749.s001.pdf]

Review of PCOMPBIOL-D-24-00885, "Aggregating multiple test results to improve medical decision-making" by Böttcher et al.

The manuscript "Aggregating multiple test results to improve medical decision-making" aims to "develop a statistical model to study how medical decision-making can be improved by repeating diagnostic and screening tests, and aggregating their results." A core theme of this work is to leverage the intuition of Boolean logic to understand and compare the performance of multiple testing approaches based on AND and OR type interpretations of results under both parallel (all tests performed simultaneously) and series (test performed sequentially with stopping criteria based on previous results) protocols. In a related vein, the manuscript considers the relative cost-savings of using series or parallel testing, and provides formulas that generalize the Rogan–Gladen estimator of prevalence.

Overall I find this manuscript to be well-written. The use of Boolean logic is a refreshing perspective on a field that, in my professional opinion, has suffered from dated perspectives. The results are often intuitive but still deserving of the rigorous attention provided in this manuscript. They also point to several deeper and interesting questions at the interface of mathematics, biology, and epidemiology.

While the manuscript is definitely worthy of publication, I do have concerns about certain parts of the exposition. I recommend revision as discussed below. Only a few of the issues I mention are major (but tractable). Most are minor suggestions that would improve the overall clarity of the manuscript. In the spirit of scientific dialogue, I would also like to point to several questions / topics that the authors might consider for the discussion section or other parts of the manuscript, as appropriate.

Overall observations on writing, structure, content

- Paper is well motivated, results likely to be useful
- Math appears correct, results generally well explained, with the exception of a few assumptions and word choices.
- Organization is excellent. Starting with a 2-test case and generalizing is a natural way to both study and write about this topic.
- The assumptions applied to specific examples are perhaps my biggest concern, and I point to this issue in my major comments below.

Major comments

1. The independence assumption (appearing around line 150) is central to the analysis, but it's also a very strong assumption. The authors should highlight the benefits and consequences of this assumption, preferably in the context of literature.

To be more specific, there are known situations wherein tests are expected to be highly correlated or anti-correlated. Take, for example, the case of a SARS-CoV-2 qPCR test and an IgG antibody test for the SARS-CoV-2 spike protein. It is well-known that the IgG response can take weeks to appear after infection, at which point a qPCR test has a high probability of being negative (because the infection has cleared). Early in the pandemic, it was generally uncommon to have a positive qPCR test and postive IgG test at the same time. So in May 2020, someone with a positive COVID-19 qPCR test was likely to be negative for SARS-CoV-2 IgG antibodies. A similar phenomenon goes for IgM and IgG. One usually precedes the other, and IgM titers wane much faster than IgG titers. (This phenomenon is common to many diseases.) This is well documented in the literature.

To this point, the authors state, "For example, the assays used in Elecsys Anti-SARS-CoV-2 (Elecsys-N) and Elecsys Anti-SARS-CoV-2 S (Elecsys-S) target antibodies against different proteins of the virus, nucleocapsid and spike proteins, respectively, so that test interactions can be neglected." Here I disagree with the spirit of what the authors are saying relative to their work. It may be the case that the *chemical interactions* in the assay between N and S are negligible, *but that does not mean there are no correlations between the antibody levels (and thus diagnostic results) in a purely probabilistic sense.* Human immune responses tend to favor the creation of both types of antibodies simultaneously, so that high N titers suggests high probability of high S titers, and thus positive test result for both. The issue is actually more important for receptor binding domain (RBD) and spike measurements, which are known to be highly correlated. Several of the works cited by the authors consider variations on these issues, and at a minimum, this paragraph needs to be fixed / clarified. (The qPCR / antibody test is also a situation where I suspect at OR protocol is best....)

Another situation worth considering is when two version of the same disease are circulating (e.g. influenza A versus B). In this case, you could have a diagnostic test tailored to one or the other, but your goal is just to determine if someone has influenza. This is a slight violation of other assumptions in the analysis, and I suspect that the "effective" TPR for detecting "just influenza" (agnostic to flu A or flu B) would be $\leq 50\%$ for one of the tests depending on the relative prevalence of A to B. But this might be a situation where independence is reasonable.

Another situation where independence might be reasonable is a "symptoms-based" diagnostic where the probability of having one symptom (e.g. a cough) is independent of the probability of having another (e.g. sore throat).

More generally, if a diagnostic is detecting a property of a pathogen (or a highly choreographed immune response), I expect correlation (or anti-correlation) in diagnostic outcomes. Physically speaking, "if you've got the pathogen, you have all parts of it at the same time." But if a diagnostic detects a physiolog-

ical response that is only indirectly tied to the pathogen and highly variable, independence is more likely.

From a mathematical standpoint it clearly makes sense to consider independent tests first. It is an easier problem on top of which independence can be built.

I recommend these issues be discussed in detail, preferably with additional examples that illustrate when independence is and is not applicable.

2. Equation 10 (for example) is a nice result that is also intuitive. But it begs several questions that I think the authors should at least consider / mention in the discussion. Clearly not all of the interpretations (or aggregated results, to use the authors' language) are meaningful. For example, if I have two tests, in many cases it's probably a bad idea to assign any combination of outcomes $[(++),(+-),(-+),(--)]$ to the negative class.... Why do the test in that case? So this leads me to the following questions.

- Does the Boolean perspective motivate a set of "admissible" interpretations of a pair of diagnostic test that does not correspond to all 16 boolean gates? If so, what are the admissible interpretations and why?
- If the admissible interpretations are a proper subset of all interpretations, does Eq. (10) or some variation thereof provide upper and lower bounds on all such interpretations? I suspect there is a more general result hiding in Eq. (10). This appears related to the discussion around lines 365 to lines 375.
- How would correlations between diagnostic tests (see above comment) affect the inequalities?

3. Equations 18 and 20. These look like harmonic means. Is there a deeper reason, or is this coincidence?

4. Table I, I'm a little wary of this example as a motivation for the following sensitivity and specificity calculations. I agree that overall COVID-19 is a motivator for this study, but for reasons in my comment 1., I'm not sure that one can defend the claim that the computations done in this section are directly applicable to those testing protocols.

5. Again, Eqs. 25, 26, independence is a major assumption here. I would remind the reader at this point of that assumption.

6. Equations 28, 29 this is in the spirit of comment 2. above. Here you have some "intermediate" set of interpretations, albeit defined in terms of the majority positive criterion. It would be nice if there were some kind of inequality bounding the sensitivities, etc. in terms of the OR and AND interpretations. Do the authors have insight on the existence of such inequalities?

7. Line 343, again I'm wary of SARS-CoV-2 testing as a justification for the $n = 3$ case. Perhaps just remove the motivation part of this sentence.

8. Line 380, where time is introduced. A key problem, e.g. in SARS-CoV-2 testing is the fact that antibody levels decay with time. Thus, it's known that the sensitivity and specificity of a test are also time-dependent. This point needs to be mentioned in the context of the discussion around line 380. This issue is more general than just COVID-19 testing. Similar issues arise in other diseases, e.g. RSV. Here the authors need to state that they are considering steady-state sensitivity and specificity.

9. Line 410, again I'm wary of this example as corresponding to independent tests. I think that assumption is violated here. As a proof of concept, the example is fine, but if the authors choose to keep the example they need to indicate that the actual tests may violate the assumptions of their analysis.

10. Equations 31 and 32 take a bit of time to work through. It might be helpful to provide a bit more justification, although the equations ultimately look correct to me. Also, see minor comment 5 below.

11. Algorithm 1 ultimately seems unneeded / overkill for this work. On line 336 the authors state that they consider all pairs $(\mathrm{TPR}_{\mathcal{S}}, \mathrm{TNR}_{\mathcal{S}})$. It looks like the convex hull is just being used to construct a reasonable interpolation of this data? Is that not correct? If so, what purpose does that actually serve? This looks like a discrete optimization problem to me, so, e.g. in the $n = 3$ case, you can directly identify the interpretation of the data that is "optimal." There doesn't seem to be a "continuous" interpretation of the assay results, so I simply don't understand what the convex hull is doing. My suggestions is that the authors clarify the exposition of the algorithm based on my comments, or else remove the algorithm if it's not needed.

Minor Comments

0. The references are a nice resource and cover a range of works that have addressed related questions. For completeness, I would suggest adding a reference by David and Johannes Brust, e.g. "Effective matrix designs for COVID-19 group testing." (in BMC Bioinformations)

1. Some of the language surrounding the use of Boolean logic is a bit confusing to me. I come from a mathematical background and am self-taught in Boolean logic. I suspect less informed readers would be confused. A few suggestions to address this.

- I would recommend including a reference to some introductory text on logic in case a reader requires some additional background.
- The paragraph starting on Line 124 is a specific example where I needed some time to parse the meaning. The first sentence, "For $n = 2$ ordered tests, there are $r = 2^n = 2^2 = 4$ possible **sequences** of test results," is clear. But the statement, "... the number of possible output **sequences** that one can assign to each of the $r = 4$ input configurations is $2^r = 16$, which is equivalent to ..., " took me some time to parse. Part of

the confusion is repetition of the word “sequences” combined with the phrase “output sequence.” It’s not immediately clear that the “output” actually corresponds to a protocol for interpreting the test results. [The word **sequence** is also confusing in light of the concept of a “series” test that comes later.] By figure 1 this started to become clear, but I had some lingering confusion. By my understanding, the statement about “number of two-input Boolean gates” at the end of that paragraph really corresponds to the number of ways or “rules” for interpreting the tests. If so, I would recommend being explicit about this, especially because not all such rules are really meaningful (e.g. always interpret the test as negative). In other words, give a “physical” explanation for what the Boolean gates correspond to.

- In a related vein, I suggest making it clear early on that not all of the gates are physically meaningful. See also my major comment 2 above.
- Similar to above, in the introduction (around line 81), the word “aggregation” is used a few times. I don’t think this is the best word choice, as the connotation is more that of “collecting” as opposed to “interpreting.” I would suggest harmonizing language between the intro and lines 124. The point really is that how one uses (i.e. interprets) the test result is subjective, whereas aggregation and boolean gating are nominally objective tasks. Similar comment line 110, line 117, etc. (look for other places where aggregate is used.) If you want to keep using aggregate, be explicit in the first few instances that this corresponds to an interpretation of all test results taken together.
- Caption of Fig. 1, instead of, “... the final result will be negative ...,” I would say something like, “the assigned class” or ”assigned disease status,” or something similar. Basically, the word “result” is used in a few contexts, and “final result” and “overall result” appear to be used interchangeably, which reduces clarity a bit. Opt for consistency of language.
- Equation 30, the language here about the number of mappings is much cleaner than description in terms of “sequences” in the two-test situation. Perhaps replace the language around line 124 with that around Eq. 30, keeping the latter as is.

2. Around line 121, define the $\Pr(X|Y)$ notation. Most people will know that this is conditional probability, but in the off-chance....

3. Line 119, I would say, “The *true* disease status...,” (i.e. insert “true” or something similar) to distinguish from the assigned disease status, which could be different.

4. Line 129 there is a typo in the first four words.

5. Line 319, I suspect the $\mathrm{TPR}_{\mathcal{S}}$ is supposed to be TPR_{S_j}.
