# Supplementary material for: Aggregating multiple test results to improve medical decision-making
Source: PLoS Comput Biol. 2025 Jan 7;21(1):e1012749. doi: 10.1371/journal.pcbi.1012749 (PMC11741652; doi:10.1371/journal.pcbi.1012749)
Supplement: Attachment — Submitted filename: response_letter.pdf [file pcbi.1012749.s002.pdf]

Response to Reviewers: Manuscript ID PCOMPBIOL-D-24-00885

November 15, 2024

Dear Editor and Reviewers,

Thank you for the careful consideration of our manuscript *Aggregating multiple test results to improve medical decision-making* and the detailed and thoughtful comments and suggestions. Here, and in the revised manuscript, we address all the reviewers' comments and requests, resulting in a much improved version.

The main changes are:

- We carefully revised all paragraphs related to test dependencies.
- In the revised Materials and Methods section, we analyzed known empirical results from multiple antibody tests to provide an example of how to quantify test dependencies.
- In the revised Materials and Methods section, we introduced a new subsection focusing on Boole–Fréchet inequalities that establish bounds on the sensitivities and specificities of several Boolean functions.
- We also revised several other sections of the manuscript to improve the exposition of our results.

Sincerely,

Lucas Böttcher, Maria R. D'Orsogna, and Tom Chou

Point-by-point discussion

Reviewer #1

Reviewer #1: The manuscript "Aggregating multiple test results to improve medical decision-making" aims to "develop a statistical model to study how medical decision-making can be improved by repeating diagnostic and screening tests, and aggregating their results." A core theme of this work is to leverage the intuition of Boolean logic to understand and compare the performance of multiple testing approaches based on AND and OR type interpretations of results under both parallel (all tests performed simultaneously) and series (test performed sequentially with stopping criteria based on previous results) protocols. In a related vein, the manuscript considers the relative cost-savings of using series or parallel testing, and provides formulas that generalize the Rogan–Gladen estimator of prevalence.

Overall I find this manuscript to be well-written. The use of Boolean logic is a refreshing perspective on a field that, in my professional opinion, has suffered from dated perspectives. The results are often intuitive but still deserving of the rigorous attention provided in this manuscript. They also point to several deeper and interesting questions at the interface of mathematics, biology, and epidemiology.

While the manuscript is definitely worthy of publication, I do have concerns about certain parts of the exposition. I recommend revision as discussed below. Only a few of the issues I mention are major (but tractable). Most are minor suggestions that would improve the overall clarity of the manuscript. In the spirit of scientific dialogue, I would also like to point to several questions/topics that the authors might consider for the discussion section or other parts of the manuscript, as appropriate.

Overall observations on writing, structure, content

- Paper is well motivated, results likely to be useful.
- Math appears correct, results generally well explained, with the exception of a few assumptions and word choices.
- Organization is excellent. Starting with a 2-test case and generalizing is a natural way to both study and write about this topic.
- The assumptions applied to specific examples are perhaps my biggest concern, and I point to this issue in my major comments below.

Authors: We are very grateful for the detailed and constructive comments from reviewer #1. We appreciate the positive feedback on our work and thank the reviewer for helping us clarify our assumptions.

Reviewer #1: **Major comments**

1. The independence assumption (appearing around line 150) is central to the analysis, but it's also a very strong assumption. The authors should highlight the benefits and consequences of this assumption, preferably in the context of literature.

To be more specific, there are known situations wherein tests are expected to be highly correlated or anti-correlated. Take, for example, the case of a SARS-CoV-2 qPCR test and an IgG antibody test for the SARS-CoV-2 spike protein. It is well-known that the IgG response can take weeks to appear after infection, at which point a qPCR test has a high probability of being negative (because the infection has cleared). Early in the pandemic, it was generally uncommon to have a positive qPCR test and postive IgG test at the same time. So in May 2020, someone with a positive COVID-19 qPCR test was likely to be negative for SARS-CoV-2 IgG antibodies. A similar phenomenon goes for IgM and IgG. One usually precedes the other, and IgM titers wane much faster than IgG titers. (This phenomenon is common to many diseases.) This is well documented in the literature.

To this point, the authors state, "For example, the assays used in Elecsys Anti-SARS-CoV-2 (Elecsys-N) and Elecsys Anti-SARS-CoV-2 S (Elecsys-S) target antibodies against different proteins of the virus, nucleocapsid and spike proteins, respectively, so that test interactions can be neglected." Here I disagree with the spirit of what the authors are saying relative to their work. It may be the case that the chemical interactions in the assay between N and S are negligible, *but that does not mean there are no correlations between the anti-body levels (and thus diagnostic results) in a purely probabilistic sense*. Human immune responses tend to favor the creation of both types of antibodies simultaneously, so that high N titers suggests high probability of high S titers, and thus positive test result for both. The issue is actually more important for receptor binding domain (RBD) and spike measurements, which are known to be highly correlated. Several of the works cited by the authors consider variations on these issues, and at a minimum, this paragraph needs to be fixed/clarified. (The qPCR / antibody test is also a situation where I suspect at OR protocol is best....)

Another situation worth considering is when two version of the same disease are circulating (e.g., influenza A versus B). In this case, you could have a diagnostic test tailored to one or the other, but your goal is just to determine if someone has influenza. This is a slight violation of other assumptions in the analysis, and I suspect that the "effective" TPR for detecting "just influenza" (agnostic to flu A or flu B) would be $\leq 50\%$ for one of the tests depending on the relative prevalence of A to B. But this might be a situation where independence is reasonable.

Another situation where independence might be reasonable is a "symptoms-based" diagnostic where the probability of having one symptom (e.g., a cough) is independent of the probability of having another (e.g., sore throat).

More generally, if a diagnostic is detecting a property of a pathogen (or a highly choreographed immune response), I expect correlation (or anti-correlation) in diagnostic outcomes. Physically speaking, "if you've got the pathogen, you have all parts of it at the same time." But if a diagnostic detects a physiological response that is only indirectly tied to the pathogen and highly variable, independence is more likely.

From a mathematical standpoint it clearly makes sense to consider independent tests first. It is an easier problem on top of which independence can be built.

I recommend these issues be discussed in detail, preferably with additional examples that illustrate when independence is and is not applicable.

Authors: We apologize for any confusion caused by our "naive Bayes" approach and corresponding statements on conditional independence. Reviewer #1 is correct in noting that our approach does not capture conditional dependencies that can arise in various testing scenarios. The assumption of conditional independence, as the reviewer also highlights, simplifies the mathematical derivation of properties of combined tests, and is one of the main reasons we used it in our original manuscript. This is consistent with what is done in most studies on medical decision-making. Additionally, manufacturers usually report performance measures for individual tests, without addressing potential dependencies between them.

We now go beyond the assumption of conditional independence by adding several discussions on conditional dependence in the revised manuscript. Specifically, we revised and extended the paragraph on test dependencies in the section "Aggregating results from two tests" and added a new section "Dependence factors" in the revised Materials and methods section. In the latter, we introduce a measure that is 1 when tests are conditionally independent, but deviates from 1 for conditionally dependent tests. The section also includes examples of results obtained from multiple antibody tests conducted on the same sample. We evaluate the dependence factor on these test results and show that in some cases it can be substantially larger than 1, indicating conditional dependence [1]. (The manufacturers were anonymized in the original study.)

Actions taken:

- In the revised section "Aggregating results from two tests", we removed our statements of independence of the two Elecsys tests and extended our discussion on test dependencies based on the feedback provided by reviewer #1: "This assumption is commonly made in the medical decision-making literature because it simplifies the mathematical analysis of aggregated test results, and manufacturers usually report performance measures for individual tests without specifying potential dependencies between them. However, test results may actually be correlated or anti-correlated. For instance, consider a SARS-CoV-2 PCR test and an IgG antibody test for the virus' spike protein. The IgG response can take weeks to develop after infection [1], by which time the PCR test is likely to return a negative result since the infection may have cleared. An example where approximate independence might hold is in symptom-based diagnostics that assess different aspects of the same disease. For instance, in some viral infections, the occurrence of fever and loss of taste may show a low degree of dependence, though this can vary depending on the disease and the underlying physiological processes. In the Materials and Methods section, we use a dataset containing the test results from nine antibody assays [1] to quantify the level of dependence between them." and "As in the derivations for the AND protocol, we assume that test results are independent. An application of the OR protocol might be testing for a disease when two versions of the same disease are circulating (*e.g.*, influenza A and B). In this case, one may use a diagnostic test specific to each strain but the goal is simply to determine if the person has influenza (*i.e.*, if either the first *or* the second test is positive)."
- In the revised Materials and Methods section, we added the new section entitled "Dependence factors" that focuses on quantifying the dependencies between test results. The dependence factor we introduce is 1 if tests are conditionally independent and deviates from 1 otherwise. We evaluate this measure on a specific example of antibody test results obtained from multiple tests conducted on the same samples and show that in some cases the dependence factor is substantially larger than 1.
- In the revised sections "Aggregating results from two tests" and "Aggregating results from more than two tests", we emphasized that generalized bounds accounting for dependence effects can be derived using the Boole–Fréchet inequalities (see our response to the second comment of reviewer #1).
- In the revised Materials and methods section, we also added a new section "Boole–Fréchet inequalities", which details the inequalities for sensitivities and specificities associated with several Boolean functions (see our response to the second comment of reviewer #1).

Reviewer #1: 2. Equation 10 (for example) is a nice result that is also intuitive. But it begs several questions that I think the authors should at least consider/mention in the discussion. Clearly not all of the interpretations (or aggregated results, to use the authors' language) are meaningful. For example, if I have two tests, in many cases it's probably a bad idea to assign any combination of outcomes [(+ +),(+ -),(- +),(- -)] to the negative class.... Why do the test in that case? So this leads me to the following questions.

- Does the Boolean perspective motivate a set of "admissible" interpretations of a pair of diagnostic test that does not correspond to all 16 boolean gates? If so, what are the admissible interpretations and why?
- If the admissible interpretations are a proper subset of all interpretations, does Eq. (10) or some variation thereof provide upper and lower bounds on all such interpretations? I suspect there is a more general result hiding in Eq. (10). This appears related to the discussion around lines 365 to lines 375.
- How would correlations between diagnostic tests (see above comment) affect the inequalities?

Authors: We thank reviewer #1 for these very relevant comments.

(1) A reformulation of the first question of reviewer #1 is: For a given pair of tests, which combined tests lie on the ROC frontier? Assuming all individual tests have discriminatory power (*i.e.*, they perform better than a random classifier), the "admissible" tests are the AND, OR, and single tests, neglecting gates that always return 0 or 1. These are the only tests that do not rely on negating any input result (see, *e.g.*, `https://mathcenter.oxford.emory.edu/site/cs170/gates/`). One can show that the ROC frontier is formed by AND, OR, and one of the single tests [8].

(2) Yes, as mentioned in our response to point (1), the ROC frontier for two tests will be formed by AND, OR, and one of the single tests.

(3) We appreciate that reviewer #1 brought up this point. If the test combinations exhibit conditional dependencies, the Boole–Fréchet inequalities [3, 9, 10, 13] can be used to establish lower and upper bounds for the sensitivities and specificities. These bounds are the best if nothing is known about the tests except their individual sensitivities and specificities. The standard Boole–Fréchet inequalities apply to AND and OR functions. One of our goals for future work is to derive inequalities that are applicable to other aggregation functions.

We clarified these points in the revised manuscript.

Actions taken:

- In the revised section "Aggregating results from two tests", we clarified point (1) of the above reviewer comment: "Assuming all individual tests have "discriminatory power" (*i.e.*, perform better than a random classifier), one can show that the set of efficient tests is formed by AND, OR, and one of the single tests [8]. A test is considered efficient if no other test performs better in one aspect (sensitivity or specificity) without performing worse in the other."
- In the revised sections "Aggregating results from two tests" and "Aggregating results from more than two tests", we emphasized that generalized bounds accounting for dependence effects can be derived using the Boole–Fréchet inequalities: "Furthermore, in the presence of dependence effects between the results of individual tests, we explain in the Materials and Methods section how to use Boole–Fréchet inequalities to formulate tight bounds that relate the sensitivities and specificities of AND and OR aggregations to those of the individual tests." and "If the results of individual tests exhibit dependence effects, bounds relating the sensitivities and specificities of several aggregation functions can be derived using the Boole–Fréchet inequalities (see Materials and methods for further details)."
- In the revised Materials and Methods section, we added a new section "Boole–Fréchet inequalities", which details the inequalities for sensitivities and specificities associated with AND, OR, and majority functions for n-test combinations.

> *Reviewer #1*: 3. Equations 18 and 20. These look like harmonic means. Is there a deeper reason, or is this coincidence?

Authors: These inequalities can indeed be expressed with harmonic means on both sides, though this appears to be coincidental.

> *Reviewer #1*: 4. Table I, I'm a little wary of this example as a motivation for the following sensitivity and specificity calculations. I agree that overall COVID-19 is a motivator for this study, but for reasons in my comment 1., I'm not sure that one can defend the claim that the computations done in this section are directly applicable to those testing protocols.

Authors: We agree that test dependencies are likely relevant to the examples listed in Table 1. Our primary goal was to provide examples of various testing configurations (*i.e.*, different Boolean functions, varying numbers of tests, and both series and parallel processing) used during the SARS-CoV-2 pandemic. Due to the large amount of data available and its global relevance, we based our work on aspects important to the SARS-CoV-2 pandemic. With appropriate adaptations, our results may also be applicable to other diseases and future pandemics. We have clarified these points in the revised manuscript.

Actions taken:

- In the revised section "Aggregating results from more than two tests", we clarified that the test protocols in Table 1 may be associated with conditionally dependent test results. We also emphasized that Boole–Fréchet inequalities may be useful in this context to bound sensitivities and specificities: "These examples illustrate the use of various testing configurations (*i.e.*, different Boolean functions, varying numbers of tests, and both series and parallel processing) during the SARS-CoV-2 pandemic. While the results of these different tests may show dependencies, we proceed with our derivations under the assumption that the results of individual tests are independent given the disease status. If the results of individual tests exhibit dependence effects, bounds relating the sensitivities and specificities of several aggregation functions to those of the individual tests can be derived using the Boole–Fréchet inequalities (see Materials and methods for further details)."
- In the revised Materials and Methods section, we added a new section "Boole–Fréchet inequalities", which details the inequalities for sensitivities and specificities associated with AND, OR, and majority functions for n-test combinations (see our response to the second comment of reviewer #1).

Reviewer #1: 5. Again, Eqs. 25, 26, independence is a major assumption here. I would remind the reader at this point of that assumption.

Authors: We agree and now remind the reader that these equations are based on the assumption of conditional independence of test results.

Actions taken:

- In the revised section "Aggregating results from more than two tests", we added "..., where we assumed that the results of different tests are conditionally independent given the disease status."

Reviewer #1: 6. Equations 28, 29 this is in the spirit of comment 2. above. Here you have some "intermediate" set of interpretations, albeit defined in terms of the majority positive criterion. It would be nice if there were some kind of inequality bounding the sensitivities, etc. in terms of the OR and AND interpretations. Do the authors have insight on the existence of such inequalities?

Authors: The majority function is intermediate to the extremes of requiring all tests to be positive (AND) and requiring at least one positive result (OR). The corresponding TPRs will thus satisfy $\mathrm{TPR}_{1\wedge 2\wedge 3} \leq \mathrm{TPR}_{\mathrm{M}(1,2,3)} \leq \mathrm{TPR}_{1\vee 2\vee 3}$.

Recall that the sensitivities of AND, OR, and the majority function M(1, 2, 3) are

$$\mathrm{TPR}_{1\wedge 2\wedge 3} = \mathrm{TPR}_1\mathrm{TPR}_2\mathrm{TPR}_3\,, \tag{R1}$$

$$\begin{aligned}\mathrm{TPR}_{1\vee 2\vee 3} =& \mathrm{TPR}_1 + \mathrm{TPR}_2 + \mathrm{TPR}_3 - \mathrm{TPR}_1\mathrm{TPR}_2 \\ & - \mathrm{TPR}_1\mathrm{TPR}_3 - \mathrm{TPR}_2\mathrm{TPR}_3 + \mathrm{TPR}_1\mathrm{TPR}_2\mathrm{TPR}_3\,,\end{aligned} \tag{R2}$$

and

$$\mathrm{TPR}_{\mathrm{M}(1,2,3)} = \mathrm{TPR}_1\mathrm{TPR}_2 + \mathrm{TPR}_1\mathrm{TPR}_3 + \mathrm{TPR}_2\mathrm{TPR}_3 - 2\mathrm{TPR}_1\mathrm{TPR}_2\mathrm{TPR}_3\,, \tag{R3}$$

respectively.

If at least one $\text{TPR}_i = 0$ (with $i \in \{1,2,3\}$), it is straightforward to show that $\text{TPR}_{1\wedge 2\wedge 3} \leq \text{TPR}_{\text{M}(1,2,3)} \leq \text{TPR}_{1\vee 2\vee 3}$.

We thus focus on the case where $\text{TPR}_i \in (0,1]$. Starting from $\text{TPR}_{1\wedge 2\wedge 3} \leq \text{TPR}_{\text{M}(1,2,3)}$, we obtain the following inequality

$$3 \leq \frac{1}{\text{TPR}_1} + \frac{1}{\text{TPR}_2} + \frac{1}{\text{TPR}_3}\,, \tag{R4}$$

which is satisfied since $\text{TPR}_i \in (0,1]$. Similarly, one can show that $\text{TPR}_{\text{M}(1,2,3)} \leq \text{TPR}_{1\vee 2\vee 3}$.

The same line of reasoning applies to the sensitivities, with $\text{TNR}_{1\wedge 2\wedge 3}$ becoming the upper bound and $\text{TNR}_{1\vee 2\vee 3}$ as the lower bound.

Actions taken:

- In the revised section "Aggregating results from more than two tests", we added the inequality reviewer #1 mentioned in the above comment: "The majority function balances between the extremes of requiring all tests to be positive (AND) and requiring at least one positive result (OR). Hence, the quantities $\text{TPR}_{\text{M}(1,2,3)}$ and $\text{TNR}_{\text{M}(1,2,3)}$ are bounded by the sensitivities and specificities of the AND and OR aggregations according to

  $$\text{TPR}_{1\wedge 2\wedge 3} \leq \text{TPR}_{\text{M}(1,2,3)} \leq \text{TPR}_{1\vee 2\vee 3} \quad \text{and} \quad \text{TNR}_{1\vee 2\vee 3} \leq \text{TNR}_{\text{M}(1,2,3)} \leq \text{TNR}_{1\wedge 2\wedge 3}\,. \tag{R5}$$

  "

Reviewer #1: 7. Line 343, again I'm wary of SARS-CoV-2 testing as a justification for the $n = 3$ case. Perhaps just remove the motivation part of this sentence.

Authors: Ww now highlight the possibility that the conditional independence assumption may not hold for these tests (see also our response to the related comment 9 of reviewer #1).

Actions taken:

- We revised the first paragraph of the section "An example with three antigen tests": "We now show how Algorithm 1 can identify efficient aggregation protocols, using an example with $n = 3$ tests. The sensitivities and specificities are based on commonly used SARS-CoV-2 antigen tests [7]. However, depending on the characteristics of these tests, the assumption of conditional independence of test results given disease status may not hold."

Reviewer #1: 8. Line 380, where time is introduced. A key problem, e.g., in SARS-CoV-2 testing is the fact that antibody levels decay with time. Thus, it's known that the sensitivity and specificity of a test are also time-dependent. This point needs to be mentioned in the context of the discussion around line 380. This issue is more general than just COVID-19 testing. Similar issues arise in other diseases, e.g., RSV. Here the authors need to state that they are considering steady-state sensitivity and specificity.

Authors: Thanks for pointing this out. We now emphasize antibody waning in the revised section "Estimating prevalence".

Actions taken:

- In the revised section "Estimating prevalence", we clarified the effect of antibody waning on test characteristics: "Additionally, depending on the test type and the disease being considered, it may be necessary to account for time-dependent sensitivities and specificities. For instance, in the context of SARS-CoV-2 tests, antibody waning is known to affect test characteristics over time [16]. In the examples we consider in this work, we assume that sensitivities and specificities remain constant."

Reviewer #1: 9. Line 410, again I'm wary of this example as corresponding to independent tests. I think that assumption is violated here. As a proof of concept, the example is fine, but if the authors choose to keep the example they need to indicate that the actual tests may violate the assumptions of their analysis.

Authors: We agree with reviewer #1 that we should acknowledge the possibility that the conditional independence assumption may not hold. As the reviewer noted, we intend to use this example as a proof of concept to demonstrate how our method can be applied in practice. It also shows the importance of empirically assessing dependence effects.

Actions taken:

- In the revised section "Correcting test errors", we acknowledge the possibility that the conditional independence assumption may not hold: "Our primary goal in this example is to use it as a proof of concept to demonstrate how errors from combined tests can be corrected in the corresponding prevalence estimates. However, depending on the properties of the two antibody tests, the assumption of conditional independence of test results given disease status may not hold."

Reviewer #1: 10. Equations 31 and 32 take a bit of time to work through. It might be helpful to provide a bit more justification, although the equations ultimately look correct to me. Also, see minor comment 5 below.

Authors: We agree with reviewer #1 that a few additional comments would help clarify this part of the manuscript. We also corrected the typo mentioned in minor comment 5.

Actions taken:

- We clarified that these equations (in particular the product $\prod_{i=1}^{n}[\mathrm{TPR}_i\delta_{i,+} + (1-\mathrm{TPR}_i)\delta_{i,-}]$) arise from the assumption of conditional independence.
- We also better highlighted our simple $n = 2$ test example that helps explain both equations.
- We replaced $\mathrm{TPR}_{\mathcal{S}}$ with $\mathrm{TPR}_{\mathcal{S}_j}$ as mentioned in minor comment 5 of reviewer #1.

Reviewer #1: 11. Algorithm 1 ultimately seems unneeded / overkill for this work. On line 336 the authors state that they consider all pairs $(\mathrm{TPR}_{\mathcal{S}}, \mathrm{TNR}_{\mathcal{S}})$. It looks like the convex hull is just being used to construct a reasonable interpolation of this data? Is that not correct? If so, what purpose does that actually serve? This looks like a discrete optimization problem to me, so, e.g., in the $n = 3$ case, you can directly identify the interpretation of the data that is "optimal." There doesn't seem to be a "continuous" interpretation of the assay results, so I simply don't understand what the convex hull is doing. My suggestions is that the authors clarify the exposition of the algorithm based on my comments, or else remove the algorithm if it's not needed.

Authors: We agree that this section requires further clarification. For a given set of n individual tests, there are $m = 2^r$ possible combinations, where $r = 2^n$. Since manually computing these combinations is impractical, we employ an algorithmic evaluation. Our goal is to identify the most efficient tests based on their TPR and TNR values. These efficient tests are located on the upper part of the convex hull in ROC space (*i.e.*, TPR-FPR space) and can be identified using a convex hull algorithm. Without such an algorithm, one would have to manually compare the sensitivities and specificities of combined tests—a task that is easily handled by the convex hull method. Our plots may have unintentionally given the impression that we wanted to determine a "continuous" curve. The dashed lines in Figure 5 were intended as guides-to-the-eye. We clarified these points in the revised manuscript.

Actions taken:

- In the revised section "Aggregation and optimization", we better highlighted the purpose of using a convex-hull algorithm: "Once we have determined all pairs $(\mathrm{TPR}_\mathcal{S}, \mathrm{TNR}_\mathcal{S})$ associated with the $m = 2^r$ test aggregations, we identify the most efficient test combinations, *i.e.*, those combinations where the underlying sensitivity-specificity pairs reach the highest values. This is achieved by employing a convex-hull algorithm, such as Graham scan [11] and Quickhull [12, 2], to determine the ROC frontier in the $(\mathrm{TPR}_\mathcal{S}, 1 - \mathrm{TNR}_\mathcal{S})$ space (*i.e.*, true positive-false positive rate space). Instead of manually comparing $\mathrm{TPR}_\mathcal{S}$ and $\mathrm{TNR}_\mathcal{S}$ values for $m = 2^r$ tests, a convex-hull algorithm can efficiently perform this task and identify the tests on the ROC frontier."
- In the revised caption of Figure 5, we clarified that dashed lines are guides-to-the-eye: "The dashed lines are guides-to-the-eye connecting the tests on the ROC curve."

Reviewer #1: **Minor comments**

0. The references are a nice resource and cover a range of works that have addressed related questions. For completeness, I would suggest adding a reference by David and Johannes Brust, e.g., "Effective matrix designs for COVID-19 group testing." (in BMC Bioinformations)

Authors: Thanks for pointing out this reference to us! We added it in the revised introduction.

Actions taken:

- In the revised introduction, we added the suggested reference in the overview of pooling strategies: "Several mathematical approaches have been developed to study the optimization of both sample pooling and testing [6]. Different proposed strategies depend on test sensitivity and specificity [18], estimated prevalence [5, 15, 17], disease dynamics ..."

Reviewer #1: 1. Some of the language surrounding the use of Boolean logic is a bit confusing to me. I come from a mathematical background and am self-taught in Boolean logic. I suspect less informed readers would be confused. A few suggestions to address this

- I would recommend including a reference to some introductory text on logic in case a reader requires some additional background.
- The paragraph starting on Line 124 is a specific example where I needed some time to parse the meaning. The first sentence, "For $n = 2$ ordered tests, there are $r = 2^n = 2^2 = 4$ possible sequences of test results," is clear. But the statement, "... the number of possible output sequences that one can assign to each of the $r = 4$ input configurations is $2^r = 16$, which is equivalent to ...," took me some time to parse. Part of the confusion is repetition of the word "sequences" combined with the phrase "output sequence." It's not immediately clear that the "output" actually corresponds to a protocol for interpreting the test results. [The word sequence is also confusing in light of the concept of a "series" test that comes later.] By figure 1 this started to become clear, but I had some lingering confusion. By my understanding, the statement about "number of two-input Boolean gates" at the end of that paragraph really corresponds to the number of ways or "rules" for interpreting the tests. If so, I would recommend being explicit about this, especially because not all such rules are really meaningful (e.g., always interpret the test as negative). In other words, give a 'physical" explanation for what the Boolean gates correspond to.
- In a related vein, I suggest making it clear early on that not all of the gates are physically meaningful. See also my major comment 2 above.
- Similar to above, in the introduction (around line 81), the word "aggregation" is used a few times. I don't think this is the best word choice, as the connotation is more that of "collecting" as opposed to "interpreting." I would suggest harmonizing language between the intro and lines 124. The point really is that how one uses (i.e. interprets) the test result is subjective, whereas aggregation and boolean gating are nominally objective tasks. Similar comment line 110, line 117, etc. (look for other places where aggregate is used.) If you want to keep using aggregate, be explicit in the first few instances that this corresponds to an interpretation of all test results taken together.
- Caption of Fig. 1, instead of, "... the final result will be negative ...,". I would say something like, "the assigned class" or "assigned disease status," or something similar. Basically, the word "result" is used in a few contexts, and "final result" and "overall result" appear to be used interchangeably, which reduces clarity a bit. Opt for consistency of language.
- Equation 30, the language here about the number of mappings is much cleaner than description in terms of "sequences" in the two-test situation. Perhaps replace the language around line 124 with that around Eq. 30, keeping the latter as is.

Authors: We thank reviewer #1 for the thorough reading and detailed comments. We incorporated all suggested changes in the revised manuscript.

Actions taken:

- In the revised introduction, we included a reference to a standard textbook on Boolean algebra and logic: "In Boolean algebra [14], this corresponds to an ..."
- In the revised section "Aggregating results from two tests", we rewrote the paragraph that focuses on the $n = 2$ test example: "For $n = 2$ ordered tests, there are $r = 2^n = 2^2 = 4$ possible sequences of test results (the permutations of "+" and "−" of length 2): $(+, +)$, $(+, -)$, $(-, +)$, and $(-, -)$. Each of these four input sequences can be mapped to either a positive ("+") or negative ("−") assigned disease status. Therefore, there are $2^r = 2^4 = 16$ possible mappings, which corresponds to the total number of two-input Boolean gates. ..."
- In the revised section "Aggregating results from two tests", we clarified that not all gates are meaningful in the context of medical decision-making: "Notice that not all gates are relevant in the context of medical decision-making. For instance, gates that return only positive or negative results would not be practical. ..."
- In the revised introduction, we clarified the term "aggregate": "We use the term "aggregate" to refer to the process of mapping multiple binary test results to a single binary output with Boolean functions."
- In the revised section "Aggregating results from two tests", we replaced "final test result" and "overall result" with "assigned disease status" to improve clarity.

> *Reviewer #1*: 2. Around line 121, define the $\Pr(X|Y)$ notation. Most people will know that this is conditional probability, but in the off-chance...

Authors: This is a very good suggestion. We defined the conditional probability $\Pr(Y \mid X)$ in the beginning of the revised section "Aggregating results from two tests".

Actions taken:

- We added the following sentence in the beginning of the revised section "Aggregating results from two tests": "We use $\Pr(Y \mid X)$ to denote the conditional probability of Y given X."

> *Reviewer #1*: 3. Line 119, I would say, "The *true* disease status...," (i.e. insert "true" or something similar) to distinguish from the assigned disease status, which could be different.

Authors: Thank you for this suggestion! We have incorporated it into the revised manuscript.

Actions taken:

- In the revised section "Aggregating results from two tests", we replaced "The disease status of an individual ..." with "The true disease status of an individual ...".

> *Reviewer #1*: 4. Line 129 there is a typo in the first four words.

Authors: We corrected this typo in the revised manuscript.

Actions taken:

- In the revised section "Aggregating results from two tests", we replaced "In Figure 1 shows two ..." with "In Figure 1, we show two ...".

Reviewer #1: 5. Line 319, I suspect the TPR_S is supposed to be TPR_{S_j} .

Authors: Thanks for spotting this typo! We corrected it in the revised manuscript.

Actions taken:

- In the revised section "Aggregation and optimization", we replaced TPR_S with TPR_{S_j} as described in the above comment of reviewer #1.

Reviewer #2

Reviewer #2: The paper discusses a statistical model designed to enhance medical decision-making by addressing uncertainties that arise from diagnostic errors—specifically, type I (false positive) and type II (false negative) errors. The model aims to improve decision accuracy by repeating and aggregating the results of diagnostic and screening tests. This methodology is applicable in both clinical settings (such as medical imaging) and public health scenarios, exemplified by the need for efficient testing during the SARS-CoV-2 pandemic.
The paper is well-written. I checked the mathematics and found it solid. However, there are some concerns as follows.

Authors: We thank reviewer #2 for their summary of our work and for the specific comments provided below. We appreciate the reviewer's positive feedback on the quality of our writing.

Reviewer #2: Practical implementation of the model in real-world settings might be complex and require substantial computational resources. The authors may want to explain this with empirical data.

Authors: Our primary motivation for this manuscript arose from discussions with immunologists and public-health experts, who emphasized the numerous ad-hoc testing protocols used during the SARS-CoV-2 pandemic were not adequately accounted for in, *e.g.*, prevalence estimates. The methods we developed address this practical need, allowing test characteristics provided by manufacturers to be easily incorporated into our model. We have also made all our source code publicly available for use by other groups [4].

We examined several examples involving the aggregation of SARS-CoV-2 test results, all of which were run on a basic Lenovo laptop, without requiring substantial computational resources. For organizations aiming to compare aggregation functions across a large number of individual tests, the number of possible combinations must indeed be considered. While this can require more substantial computations (for quantifying the characteristics of aggregated test results), this is a natural consequence of the underlying combinatorial optimization problem.

In the revised manuscript, we have added a new "real-world" example where results from empirical antibody tests conducted on the same samples were made publicly available [1]. We used this example to illustrate how empirical test data can be used to quantify test dependencies and produce more accurate estimates of the characteristics of combined tests. Computations were not prohibitive and were performed on a single core of a 1.8 GHz i7 processor on a standard laptop.

Actions taken:

- In the revised section "Aggregating results from two tests", we comment on the new empirical dataset we considered: " In the Materials and methods section, we use a dataset containing the test results from nine antibody assays [1] to quantify the level of dependence between them."
- In the revised Materials and Methods section, we added the new section titled "Dependence factors", which focuses on quantifying the dependencies between test results. We use a specific example of antibody test results obtained from multiple tests conducted on the same samples.

Reviewer #2: The effectiveness of the model may depend on the availability and quality of observational data. This paper is largely theoretical without extensive experiments with real data. More experiments to demonstrate its utility is required before it can be published. Similarly, while the model is designed to be adaptable, its performance across different diseases and testing scenarios would need thorough validation.

Authors: In general, our theoretical framework advances the field by proposing various methods to (i) aggregate results from different diagnostic tests and (ii) improve estimates of key measures in infectious-disease surveillance. Tests can be combined using different Boolean functions (AND, OR, majority rule, etc.), orderings (sequential or parallel) and the number of tests can vary. Once the specificity and sensitivity of the tests are established—information usually provided by manufacturers and public-health authorities—we can determine which aggregation options are superior. This allows us to identify those that offer the highest accuracy or the greatest savings in test usage. We also showed how our methods can be used to derive prevalence estimates that account for errors associated with test protocols that involve more than one test.

We identified several concrete examples in the literature, including SARS-CoV-2 testing protocols that employ different Boolean functions and estimates of infectious-disease surveillance measures (such as prevalence, infection fatality ratio, and infection hospitalization ratio) derived from multiple tests. We were also able to show that our methods are useful in quantifying the level of conditional dependence among empirical test results from nine different antibody assays provided in [1]. Our approach enabled the extraction of meaningful measures to characterize the "interactions" between assays within this dataset. Since some of the assumptions we made may not always be applicable in "real-life", we relaxed some of them, primarily that of conditional independence, and determined upper and lower bounds on the aggregated measures.

In the revised manuscript, we also discuss the applicability of our work to cases where, for example, an individual is affected by multiple strains of the same virus, or where different tests are based on different biochemical principles and may have different response times. It is important to note that, to the best of our knowledge, aggregation of multiple test results is still an understudied topic in epidemiology and public health. Its relevance will most likely grow due to the need for accurate management of future infectious-disease outbreaks, and technological advancements. We discuss these points in the revised manuscript and hope our work stimulates further research in this direction.

Actions taken:

- In the revised section "Aggregating results from two tests", we emphasized the relevance of our results to situations where, for example, an individual is infected with multiple strains of the same virus or where different tests rely on various biochemical principles and may exhibit different response times.
- In the revised Discussion section, we commented on the practical relevance of our work and potential future extensions: "Given the growing need to effectively manage future infectious disease outbreaks and the ongoing advancements in technology, the methods presented in this work, along with potential future extensions, are likely to be valuable for improving both testing protocols and estimates of infectious-disease surveillance measures such as prevalence, infection fatality ratio, and infection hospitalization ratio."
- In the revised manuscript, we included new results to quantify the dependencies between test outcomes using a comprehensive set of observational data from nine antibody tests [1] (see our response to the prior comment of reviewer #2).

References

[1] Emily R. Adams, Mark Ainsworth, Rupa Anand, Monique I. Andersson, Kate Auckland, J. Kenneth Baillie, Eleanor Barnes, Sally Beer, James I. Bell, Thomas Berry, Shaista Bibi, Miles Carroll, Subhash K. Chinnakannan, Elizabeth Clutterbuck, Richard J. Cornall, Derrick W. Crook, Thushan I. de Silva, Wanwisa Dejnirattisai, Katie E. Dingle, Christina Dold, Ana Espinosa, David W. Eyre, Helen Farmer, Maria Fernandez Mendoza, Dimitrios Georgiou, Sarah J. Hoosdally, Andrew Hunter, Katie Jefferey, Dominic F. Kelly, Paul Klenerman, Julian Knight, Caroline Knowles, Alvin J. Kwok, Ursula Leuschner, Rachel Levin, C. Liu, Cesar López-Camacho, Jacqueline Martinez, Philip C. Matthews, Hannah McGivern, Alexander J. Mentzer, Joshua Milton, Juthathip Mongkolsapaya, Sarah C. Moore, Mariana S. Oliveira, Filipe Pereira, Emily Perez, Tim Peto, Richard J. Ploeg, Andrew Pollard, Timothy Prince, David J. Roberts, Justine K. Rudkin, Vicky Sanchez, Gavin R. Screaton, Malcolm G. Semple, Jose Slon-Campos, Daniel T. Skelly, Eleanor N. Smith, Andrew Sobrinodiaz, Jackie Staves, David I. Stuart, Piyada Supasa, Tony Surik, Hayley Thraves, Philip Tsang, Lance Turtle, A. Sarah Walker, Barbara Wang, Cheryl Washington, Natalie Watkins, James Whitehouse, and National COVID Testing Scientific Advisory Panel. Antibody testing for COVID-19: A report from the National COVID Scientific Advisory Panel. *Wellcome Open Research*, 5:139, 2021.

[2] C Bradford Barber, David P Dobkin, and Hannu Huhdanpaa. The quickhull algorithm for convex hulls. *ACM Transactions on Mathematical Software*, 22(4):469–483, 1996.

[3] George Boole. *An Investigation of the Laws of Thought, On Which Are Founded the Mathematical Theories of Logic and Probability*. Walton and Maberly, London, UK, 1854.

[4] Lucas Böttcher. GitLab repository, 2024. `https://gitlab.com/ComputationalScience/testing-statistics`.

[5] David Brust and Johannes J Brust. Effective matrix designs for COVID-19 group testing. *BMC Bioinformatics*, 24(1):26, 2023.

[6] Evangeline Ann Daniel, Bennett Henzeler Esakialraj L, Anbalagan S, Kannan Muthuramalingam, Ramesh Karunaianantham, Lucia Precilla Karunakaran, Manohar Nesakumar, Murugesan Selvachithiram, Sathyamurthi Pattabiraman, Sudhakar Natarajan, Srikanth Prasad Tripathy, and Luke Elizabeth

Hanna. Pooled testing strategies for SARS-CoV-2 diagnosis: A comprehensive review. *Diagnostic Microbiology and Infectious Disease*, 101(2):115432, 2021.

[7] J. Dinnes, P. Sharma, S. Berhane, SS. van Wyk, N. Nyaaba, J. Domen, M. Taylor, J. Cunningham, C. Davenport, S. Dittrich, D. Emperador, L. Hooft, MMG. Leeflang, MDF. McInnes, R. Spijker, JY. Verbakel, Y. Takwoingi, S. Taylor-Phillips, A. Van den Bruel, and JJ. Deeks. Rapid, point-of-care antigen tests for diagnosis of SARS-CoV-2 infection. *Cochrane Database of Systematic Reviews*, 7(7), 2022.

[8] Stefan Felder and Thomas Mayrhofer. *Medical Decision Making: A Health Economic Primer, 3rd Ed.* Springer-Verlag, Berlin, Germany, 2022.

[9] Maurice Fréchet. Généralisations du théorème des probabilités totales. *Fundamenta Mathematicae*, 25:379–387, 1935.

[10] Maurice Fréchet. Sur les tableaux de corrélation dont les marges sont données. *Annales de l'Université de Lyon. Section A: Sciences mathématiques et astronomie*, 9:53–77, 1951.

[11] Ronald L. Graham. An efficient algorithm for determining the convex hull of a finite planar set. *Information Processing Letters*, 1(4):132–133, 1972.

[12] Jonathan S. Greenfield. A proof for a QuickHull algorithm. Technical report, Electrical Engineering and Computer Science, April 1990.

[13] Theodore Hailperin. Best possible inequalities for the probability of a logical function of events. *The American Mathematical Monthly*, 72(4):343–359, 1965.

[14] Alan B Marcovitz. *Introduction to Logic Design*. McGraw-Hill Higher Education, New York, NY, 3rd edition, 2010.

[15] Leon Mutesa, Pacifique Ndishimye, Yvan Butera, Jacob Souopgui, Annette Uwineza, Robert Rutayisire, Ella Larissa Ndoricimpaye, Emile Musoni, Nadine Rujeni, Thierry Nyatanyi, Edouard Ntagwabira, Muhammed Semakula, Clarisse Musanabaganwa, Daniel Nyamwasa, Maurice Ndashimye, Eva Ujeneza, Ivan Emile Mwikarago, Claude Mambo Muvunyi, Jean Baptiste Mazarati, Sabin Nsanzimana, Neil Turok, and Wilfred Ndifon. A pooled testing strategy for identifying SARS-CoV-2 at low prevalence. *Nature*, 589:276–280, 2020.

[16] Nana Owusu-Boaitey, Timothy W Russell, Gideon Meyerowitz-Katz, Andrew T Levin, and Daniel Herrera-Esposito. Dynamics of SARS-CoV-2 seroassay sensitivity: a systematic review and modelling study. *Eurosurveillance*, 28(21):2200809, 2023.

[17] Hannes Schenk, Yasemin Caf, Ludwig Knabl, Christoph Mayerhofer, and Wolfgang Rauch. High prevalence group testing in epidemiology with geometrically inspired algorithms. *Scientific Reports*, 13:18910, 2023.

[18] Marie Wunsch, Dominik Aschemeier, Eva Heger, Denise Ehrentraut, Jan Krüger, Martin Hufbauer, Adnan S Syed, Gibran Horemheb-Rubio, Felix Dewald, Irina Fish, Maike Schlotz, Henning Gruell, Max Augustin, Clara Lehmann, Rolf Kaiser, Elena Knops, Steffi Silling, and Florian Klein. Safe and effective pool testing for SARS-CoV-2 detection. *Journal of Clinical Virology*, 145:105018, 2021.
